# A Retrospective Evaluation of the Effects of Cumulative Fluid Balance on Mortality and Morbidity in Critically Ill Patients in a Tertiary Intensive Care Unit (ICU) in Brisbane, Australia

**DOI:** 10.7759/cureus.96794

**Published:** 2025-11-13

**Authors:** Ravinesh Singh, James Walsham, Kyle White, Jason Meyer, Rod Hufford

**Affiliations:** 1 Intensive Care Unit, Princess Alexandra Hospital, Brisbane, AUS

**Keywords:** critical care, cumulative fluid balance (cfb), emergency critical care, intensive care, medical intensive care unit (micu), morbidity, mortality

## Abstract

Introduction

The administration of fluid therapy and fluid management is common in the intensive care unit (ICU). There is an increasing trend toward an association between fluid balance and patient outcomes, including mortality. Despite extensive research, the best approach to fluid management remains uncertain. In this study, we tested the hypothesis that a more positive cumulative fluid balance (CFB) is associated with increased hospital mortality in ICU patients.

Design and setting

This is a single-center retrospective cohort study conducted at a tertiary ICU (PAH; Brisbane, Australia) from January 2015 to June 2021. Fourteen thousand three hundred thirty-nine admissions were screened, from which 2,392 individual patient admissions were analyzed.

Main outcome measures

Patients were grouped into quintiles based on 120 hours of CFB. This time frame was defined a priori. Actual mortality (Intensive Care Unit and Hospital) was compared to predicted mortality (Acute Physiology and Chronic Health Evaluation (APACHE III) and Australian and New Zealand Risk of Death (ANZROD)).

Results

Hospital mortality increased progressively from 13.5% in the least fluid positive group to 38.8% in the most fluid positive group. When adjusted for disease severity scores, the actual mortality when compared to predicted outcomes was lowest in the less fluid positive quintiles and increased progressively to become greater than predicted in the most fluid positive quintile. The more fluid positive patients had higher APACHE III scores, were more likely to require higher doses of vasopressors, had more severe kidney injury, more requirement for dialysis and invasive mechanical ventilation, were more likely to be postoperative, and had a trauma diagnosis. The less fluid positive patients required lower doses of vasopressors, less invasive mandatory ventilation, and had longer ICU and hospital stays, and more furosemide use, with the diagnosis of sepsis over-represented in this group.

Conclusions

A higher CFB at 120 hours of ICU admission was associated with increased hospital mortality, even after adjustment for illness severity. These findings support the need for prospective studies to define optimal fluid strategies in critically ill patients.

## Introduction

Fluid administration is one of the most common therapies for critically ill patients and is ubiquitous in the intensive care unit (ICU). Adequate resuscitation is a fundamental part of critical care management, including fluid administration for acute circulatory failure [1,2]. Fluid therapy may be lifesaving, but for any individual patient, too much or too little fluid can be detrimental [1].

The three phases of resuscitation include: (i) rescue or resuscitation phase which involves the administration of fluid to address immediate life threats such as a shocked state with the aim of improving tissue perfusion by increasing venous return, stroke volume and therefore cardiac output and organ perfusion, (ii) titration phase which involves an adjustment of the fluid type and rate to achieve optimal tissue perfusion in a patient who is stabilizing, and (iii) de-escalation which involves minimizing fluid administration and mobilizing extra fluid in order to optimize fluid balance [3]. Therefore, CBF changes over the course of a patient’s admission to the ICU.

Critically ill patients are significantly heterogeneous, necessitating the need for a nuanced fluid strategy [4]. In addition, admission to the ICU occurs from various sources (such as the emergency department, hospital wards, operating theaters, or other hospitals), and the outcome depends on a complex interplay of various factors [5].

Observational data in the literature demonstrate an association between fluid balance and outcomes such as mortality, acute kidney injury (AKI), and need for invasive mechanical ventilation (IMV) [5,6]. However, despite extensive research, controversy remains regarding the best approach to fluid therapy, and there is no universally accepted method of reliably determining if a patient benefits from fluid administration [1].

We sought to use a large patient database to explore the association between cumulative fluid balance (CFB) and outcomes with adjustment for standardized mortality rates. We aimed to test the primary hypothesis that a more positive cumulative fluid balance is associated with increased hospital mortality in critically ill patients.

## Materials and methods

Trial design and setting

This was a retrospective, single-center cohort study of patients admitted to the Princess Alexandra Hospital (PAH; Brisbane, Australia) ICU from January 2015 to June 2021. The PAH is a 1,052-bed university-affiliated tertiary hospital with a 30-bed ICU serving all major sub-specialties except for burns and obstetrics. Quaternary services include liver and renal transplantation, spinal injuries, cardiothoracic surgery, and extracorporeal membrane oxygenation (ECMO). The study population, primary, and secondary outcomes of interest were defined prior to data acquisition and analysis. CFB was defined as being the sum of all intakes and outputs of all fluids over a set period within the ICU.

Study Population

Inclusion criteria were all patients with a length of stay (LOS) in the ICU of 120 hours or longer. Patients were excluded if they were <18 years of age or were readmissions to the ICU.

Outcomes

The primary outcome evaluated in the study was the hospital mortality rate associated with quintiles of CFB at 120 hours post-admission to the ICU. The actual mortality was compared to predicted mortality using APACHE III and Australia and New Zealand Risk of Death (ANZROD) scores. The Standardised Mortality Ratio (SMR), which demonstrates the ratio of actual mortality versus standardized expected mortality for each of the quintiles, was calculated.

The other outcomes assessed were ICU LOS, Hospital LOS, incidence and duration of IMV, noninvasive ventilation (NIV), renal replacement therapy (RRT), vasoactive support, and dose of diuretic (furosemide) delivered. Daily vasoactive support data were collected using the Vasoactive Inotropic Score (VIS), which was calculated as a weighted sum of the peak daily dose of all administered inotropes and vasoconstrictors, further explained in the Supplementary Appendix [7]. Baseline creatinine (Cr) was taken as the nadir Cr during the first 5 days, and, along with using urine output, the incidence and severity of renal failure were established according to the Kidney Disease Improving Global Outcomes (KDIGO) criteria [8].

Data sources

Data routinely collected were extracted from the ICU electronic medical record (MetaVision® Clinical Information System (CIS); iMDSoft, Tel Aviv, Israel). This included fluid balance data, which comprised all input and outputs entered into the CIS from the time of admission to the ICU, information on ventilation and RRT, vasoactive use, dosage of diuretics, and serum creatinine values. Sequential Organ Failure Assessment (SOFA) scores were calculated daily from data within the CIS, apart from central nervous system SOFA scores, which were not collected due to sedation confounding valid assessment from CIS data.

Information on baseline demographics, admission diagnosis, ICU and hospital LOS, severity of illness, and outcomes was obtained from the Australia and New Zealand Intensive Care Society (ANZICS) Centre for Outcome and Resource Evaluation (CORE) Adult Patient Database (APD). Patients who had missing data were excluded. When calculating VIS, if the patient had less than 24 hours but more than 6 hours of data, the latter was extrapolated to 24 hours.

Statistical analysis

Data was entered into the comma-separated values files with Transact-structured query language, and statistical analysis was conducted using the software R within RStudio [9,10]. CFB was calculated at 120 hours for each patient and then separated into quintiles (Q). Cut points were based on maintaining equal group size, with the first and last group absorbing the remaining two patients.

Continuous baseline characteristics were checked for normality using the Shapiro test. No variables were found to have a normal distribution, and all continuous variables were reported as median (interquartile range - IQR). Categorical data has been reported as a number (percentage). Significance tests were carried out with the Kruskal-Wallis Rank Sum Test for continuous data and the chi-squared test for categorical data.

When comparing quintiles in the primary analysis, a two-sided binomial exact test was conducted between actual mortality and the mean predicted mortality. Differences between the actual and predicted mortality rates were considered significant if the p-value was less than 0.05. Results from the binomial exact test gave the actual mortality rate with 95% confidence intervals and a p-value to indicate if the difference between the observed and predicted mortality was significant. A SMR was then calculated for ease of interpretation. All Kaplan-Meier curves were generated as risk-based curves (1 - x) and were reported without adjustment.

## Results

Study cohort

**Figure 1:**
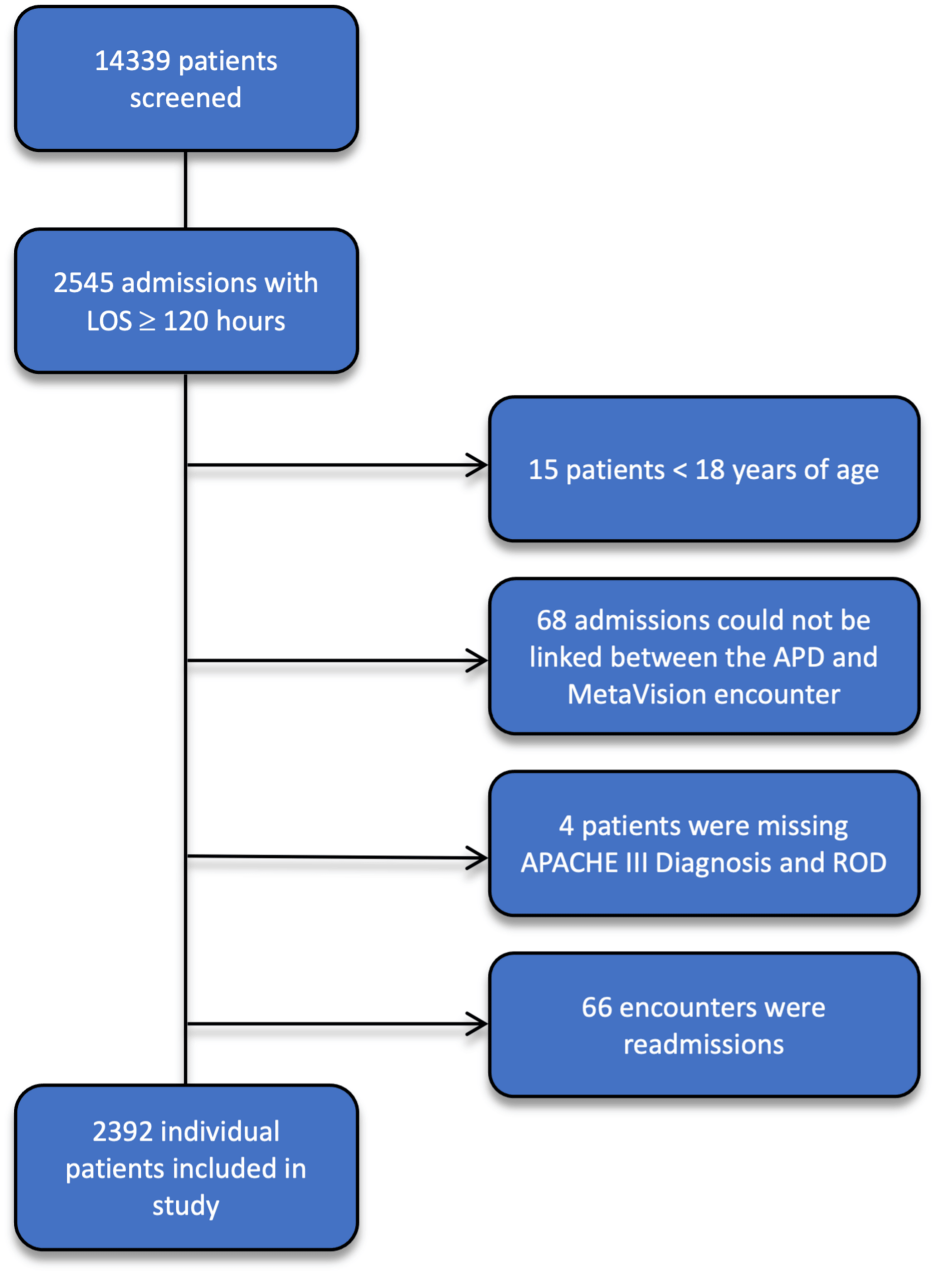
Study patient flowchart

A total of 14,339 admissions occurred during the study period. Two thousand five hundred forty-five were admitted for over 120 hours, of which 2,392 individual patient admissions were included in the study. Seventy-two patients were excluded due to missing data. Patient flow is depicted in Figure 1.

**Figure 2:**
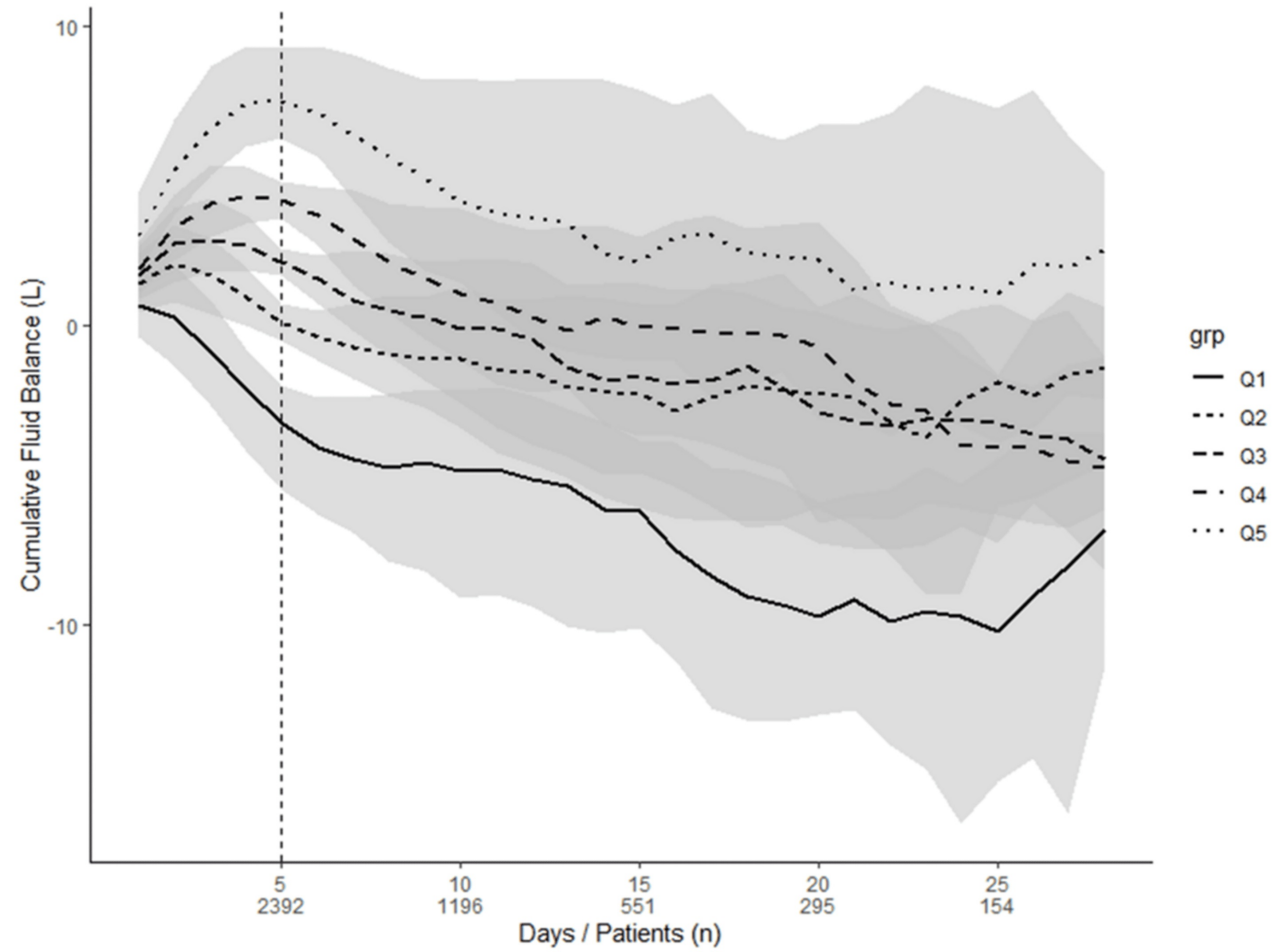
The trend of daily median cumulative fluid balance (with Interquartile range) across the quintiles over 28 days in ICU Lines represent medial values of CFB over the days. Shaded areas represent the interquartile range (IQR) between the 25th and 75th percentiles. CFB: cumulative fluid balance in liters; Q: quintile with Q1 representing the least fluid positive and Q5 the most fluid positive

The patient cohort was divided into quintiles based on their ICU CFB after 120 hours. Median CFB ranged from negative 3.26 L in Q1, representing the least fluid positive group, to positive 7.48 L in Q5, representing the most fluid positive group. Table 1 gives further data on the quintiles. Figure 2 shows the daily median and the IQR of CFB for each quintile over 28 days.

**Table 1:** Cumulative fluid balance data at 120 hours of ICU admission representing each quintile. Q: Quintile; L: Liters; IQR: interquartile range

Fluid Group	n	Median (IQR) in L	Range in L
Q1	479	-3.26 (-5.469 to -1.982)	-19.097 to -1.197
Q2	478	0.136 (-0.483 to 0.71)	-1.185 to 1.199
Q3	478	2.11 (1.711 to 2.571)	1.201 to 3.054
Q4	478	4.174 (3.588 to 4.797)	3.055 to 5.448
Q5	479	7.482 (6.28 to 9.268)	5.459 to 21.992

Baseline characteristics

The study cohort baseline characteristics are found in Table 2. There were several significant differences in the baseline characteristics of the quintiles. Patients with higher CFB at 120 hours had higher APACHE III scores, were more likely to require and receive higher doses of vasopressors. They had more severe kidney injury and a requirement for dialysis with higher rates of IMV but lower rates of NIV. The more fluid positive groups also consisted of a greater proportion of trauma and postoperative admissions.

**Table 2:** Baseline characteristics at time of study inclusion (120 hours of ICU admission) Categorical variables are number (%) and continuous variables are median (IQR) unless otherwise noted. IQR: interquartile range; BMI: body mass index; LOS: length of stay; SOFA: Sequential Organ Failure Assessment Score; APACHE: Acute Physiology and Chronic Health Evaluation, calculated on Day 1; CHS: chronic health score; IMV: invasive mechanical ventilation; NIV: noninvasive ventilation; VIS: Vasoactive Inotrope Score; KDIGO AKI: Kidney Disease Improving Global Outcomes Acute Kidney Injury stage; CRRT: continuous renal replacement therapy; Mg: milligrams; L: Liters; CVS: cardiovascular system; GI: gastrointestinal. *During the first 120 hours of admission ^#^SOFA Score does not include neurological component ^$^only includes admissions to June.

Variable	Overall	Q1	Q2	Q3	Q4	Q5	p-value
No.	2392	479	478	478	478	479	
Sex (F)	806 (33.7)	167 (34.9)	175 (36.6)	155 (32.4)	163 (34.1)	146 (30.5)	0.319
Height (cm)	172 (165 to 180)	173 (165 to 179)	170 (165 to 179)	172 (165 to 180)	172 (165 to 180)	173 (165 to 180)	0.490
Weight (kg)	82.0 (70.0, 98.0)	85.0 (70.5, 100.0)	80.0 (70.0, 95.0)	80.0 (70.0, 95.0)	80.0 (70.0, 95.0)	85.0 (72.8, 100.0)	0.003
BMI	27.7 (24.2, 32.7)	28.2 (24.7, 34.0)	27.7 (24.2, 32.0)	27.5 (23.9, 32.4)	27.7 (23.9, 31.9)	27.8 (24.5, 32.9)	0.034
Pre-ICU LOS (days)	0.25 (0.14, 1.08)	0.33 (0.13, 2.89)	0.26 (0.14, 1.24)	0.23 (0.15, 0.60)	0.24 (0.15, 0.90)	0.24 (0.16, 0.60)	0.024
APACHE III Score	69 (51 to 88)	70 (53 to 86)	66 (49 to 84)	70 (49 to 89)	68 (50 to 87)	74 (53 to 93)	0.016
Had an APACHE III Chronic Health Condition	314 (13.1)	78 (16.3)	57 (11.9)	53 (11.1)	59 (12.3)	67 (14.0)	0.131
ANZROD Risk of Death	0.141 (0.043, 0.354)	0.160 (0.055, 0.351)	0.128 (0.041, 0.315)	0.136 (0.038, 0.398)	0.138 (0.039, 0.329)	0.146 (0.044, 0.388)	0.207
Diagnostic Group							<0.001
Trauma	617 (25.8)	49 (10.2)	106 (22.2)	142 (29.7)	145 (30.3)	175 (36.5)	
CVS	516 (21.6)	113 (23.6)	107 (22.4)	108 (22.6)	103 (21.5)	85 (17.7)	
Neurological	458 (19.1)	88 (18.4)	106 (22.2)	106 (22.2)	94 (19.7)	64 (13.4)	
Sepsis	289 (12.1)	89 (18.6)	46 (9.6)	42 (8.8)	54 (11.3)	58 (12.1)	
GI	222 (9.3)	60 (12.5)	40 (8.4)	32 (6.7)	41 (8.6)	49 (10.2)	
Respiratory	175 (7.3)	55 (11.5)	41 (8.6)	33 (6.9)	30 (6.3)	16 (3.3)	
Metabolic	60 (2.5)	10 (2.1)	21 (4.4)	7 (1.5)	6 (1.3)	16 (3.3)	
Other	55 (2.3)	15 (3.1)	11 (2.3)	8 (1.7)	5 (1.0)	16 (3.3)	
Post Operative Admission	815 (34.1)	128 (26.7)	165 (34.5)	181 (37.9)	165 (34.5)	176 (36.7)	0.003
Received IMV*	2238 (93.6)	409 (85.4)	451 (94.4)	457 (95.6)	460 (96.2)	461 (96.2)	<0.001
Received NIV*	507 (21.2)	139 (29.0)	109 (22.8)	104 (21.8)	99 (20.7)	56 (11.7)	<0.001
Received Vasoactives*	2109 (88.2)	405 (84.6)	399 (83.5)	424 (88.7)	429 (89.7)	452 (94.4)	<0.001
Highest VIS*	10.96 (4.74, 21.54)	9.50 (3.33, 19.79)	8.44 (3.84, 19.74)	10.75 (4.67, 19.19)	11.83 (5.47, 20.93)	16.43 (6.84, 27.76)	<0.001
Highest KDIGO AKI Stage*							<0.001
Stage 0	447 (18.7)	115 (24.0)	116 (24.3)	103 (21.5)	72 (15.1)	41 (8.6)	
Stage 1	650 (27.2)	140 (29.2)	147 (30.8)	142 (29.7)	130 (27.2)	91 (19.0)	
Stage 2	673 (28.1)	84 (17.5)	125 (26.2)	138 (28.9)	169 (35.4)	157 (32.8)	
Stage 3	622 (26.0)	140 (29.2)	90 (18.8)	95 (19.9)	107 (22.4)	190 (39.7)	
Received CRRT*	320 (13.4)	96 (20.0)	35 (7.3)	48 (10.0)	50 (10.5)	91 (19.0)	<0.001
Highest Daily SOFA Score*^#^	8.0 (6.0, 10.3)	8.0 (6.0, 11.0)	7.0 (6.0, 10.0)	8.0 (6.0, 10.0)	8.0 (6.0, 10.0)	9.0 (7.0, 12.0)	<0.001
Highest Respiratory SOFA*							0.004
0	3 (0.1)	0 (0.0)	0 (0.0)	3 (0.6)	0 (0.0)	0 (0.0)	
1	83 (3.5)	19 (4.0)	20 (4.2)	15 (3.2)	20 (4.2)	9 (1.9)	
2	753 (31.7)	138 (29.1)	158 (33.1)	165 (34.7)	147 (30.9)	145 (30.5)	
3	1135 (47.7)	215 (45.3)	221 (46.3)	234 (49.3)	226 (47.6)	239 (50.3)	
4	403 (17.0)	103 (21.7)	78 (16.4)	58 (12.2)	82 (17.3)	82 (17.3)	
Highest Coagulopathy SOFA*							<0.001
0	1048 (44.0)	230 (48.1)	240 (50.4)	226 (47.5)	201 (42.3)	151 (31.8)	
1	630 (26.5)	102 (21.3)	120 (25.2)	127 (26.7)	145 (30.5)	136 (28.6)	
2	465 (19.5)	97 (20.3)	75 (15.8)	92 (19.3)	84 (17.7)	117 (24.6)	
3	182 (7.6)	39 (8.2)	32 (6.7)	24 (5.0)	37 (7.8)	50 (10.5)	
4	55 (2.3)	10 (2.1)	9 (1.9)	7 (1.5)	8 (1.7)	21 (4.4)	
Highest Liver SOFA*							<0.001
0	1115 (46.8)	188 (39.3)	247 (51.8)	259 (54.4)	249 (52.3)	172 (36.2)	
1	649 (27.2)	139 (29.1)	125 (26.2)	119 (25.0)	123 (25.8)	143 (30.1)	
2	456 (19.1)	104 (21.8)	78 (16.4)	78 (16.4)	81 (17.0)	115 (24.2)	
3	103 (4.3)	23 (4.8)	15 (3.1)	15 (3.2)	17 (3.6)	33 (6.9)	
4	59 (2.5)	24 (5.0)	12 (2.5)	5 (1.1)	6 (1.3)	12 (2.5)	
Highest Renal SOFA*							<0.001
0	1187 (49.6)	217 (45.3)	266 (55.6)	267 (55.9)	254 (53.1)	183 (38.2)	
1	479 (20.0)	86 (18.0)	104 (21.8)	90 (18.8)	95 (19.9)	104 (21.7)	
2	219 (9.2)	55 (11.5)	36 (7.5)	39 (8.2)	49 (10.3)	40 (8.4)	
3	116 (4.8)	19 (4.0)	26 (5.4)	25 (5.2)	15 (3.1)	31 (6.5)	
4	391 (16.3)	102 (21.3)	46 (9.6)	57 (11.9)	65 (13.6)	121 (25.3)	
Highest Cardiovascular SOFA*							<0.001
0	62 (2.6)	17 (3.5)	16 (3.3)	11 (2.3)	12 (2.5)	6 (1.3)	
1	220 (9.2)	56 (11.7)	63 (13.2)	43 (9.0)	37 (7.7)	21 (4.4)	
2	3 (0.1)	2 (0.4)	0 (0.0)	0 (0.0)	1 (0.2)	0 (0.0)	
3	476 (19.9)	102 (21.3)	107 (22.4)	104 (21.8)	80 (16.7)	83 (17.3)	
4	1631 (68.2)	302 (63.0)	292 (61.1)	320 (66.9)	348 (72.8)	369 (77.0)	
Total Furosemide Dose (mg)*	40 (0 to 160)	140 (0 to 280)	60 (0 to 180)	40 (0 to 150)	40 (0 to 120)	20 (0 to 100)	<0.001
Total Cumulative Fluid Balance (L)*	2.11 (−0.49, 4.80)	-3.26 (−5.47, −1.98)	0.14 (−0.48, 0.71)	2.11 (1.71, 2.57)	4.17 (3.59, 4.80)	7.48 (6.28, 9.27)	<0.001
Year Admitted							0.001
2015	333 (13.9)	58 (12.1)	52 (10.9)	57 (11.9)	78 (16.3)	88 (18.4)	
2016	372 (15.6)	71 (14.8)	76 (15.9)	73 (15.3)	76 (15.9)	76 (15.9)	
2017	343 (14.3)	54 (11.3)	68 (14.2)	69 (14.4)	67 (14.0)	85 (17.7)	
2018	360 (15.1)	80 (16.7)	65 (13.6)	81 (16.9)	55 (11.5)	79 (16.5)	
2019	369 (15.4)	78 (16.3)	79 (16.5)	72 (15.1)	77 (16.1)	63 (13.2)	
2020	414 (17.3)	97 (20.3)	84 (17.6)	90 (18.8)	84 (17.6)	59 (12.3)	
2021^$^	201 (8.4)	41 (8.6)	54 (11.3)	36 (7.5)	41 (8.6)	29 (6.1)	

Patients with the least positive CFB appear to have greater numbers of patients having a longer pre-ICU LOS and a greater proportion of respiratory and sepsis diagnoses but a lower proportion of trauma diagnosis. These patients also had the highest Hospital and ICU LOS (Tables 3-4). Moreover, both Q1 and Q5 had a greater incidence of AKI and requirement for RRT; however, in contrast to Q5, they received higher doses of furosemide.

**Table 3:** Hospital length of stay (in days) demonstrated across the five quintiles CFB: cumulative fluid balance; L: liters; IQR: interquartile range; LWR: lower; UPRE: upper; SD: standard deviation; Q: quintiles; LOS: length of stay; ICU: intensive care unit There has been no adjustment made in the statistical analysis for death.

CFB (L)	Mean Hospital LOS	SD	Median	Lower IQR	Upper IQR
Q1	49.67	50.84	34	20.5	57.5
Q2	45.53	54.31	27	17.0	55.0
Q3	44.11	59.77	24	14.0	49.0
Q4	41.39	45.38	25	15.0	48.0
Q5	46.78	57.94	24	12.0	57.5

**Table 4:** Intensive care unit length of stay (in days) demonstrated across the five quintiles CFB: cumulative fluid balance; L: liters; IQR: interquartile range; LWR: lower; UPRE: upper; SD: standard deviation; Q: quintiles; LOS: length of stay; ICU: intensive care unit There has been no adjustment made in the statistical analysis for death.

CFB (L)	Mean ICU LOS	SD	Median	Lower IQR	Upper IQR
Q1	16.46	15.87	13	8	19
Q2	11.83	8.34	10	7	13
Q3	9.72	5.98	8	6	11
Q4	9.84	6.61	8	6	11
Q5	11.24	7.82	9	6	13

**Figure 3:**
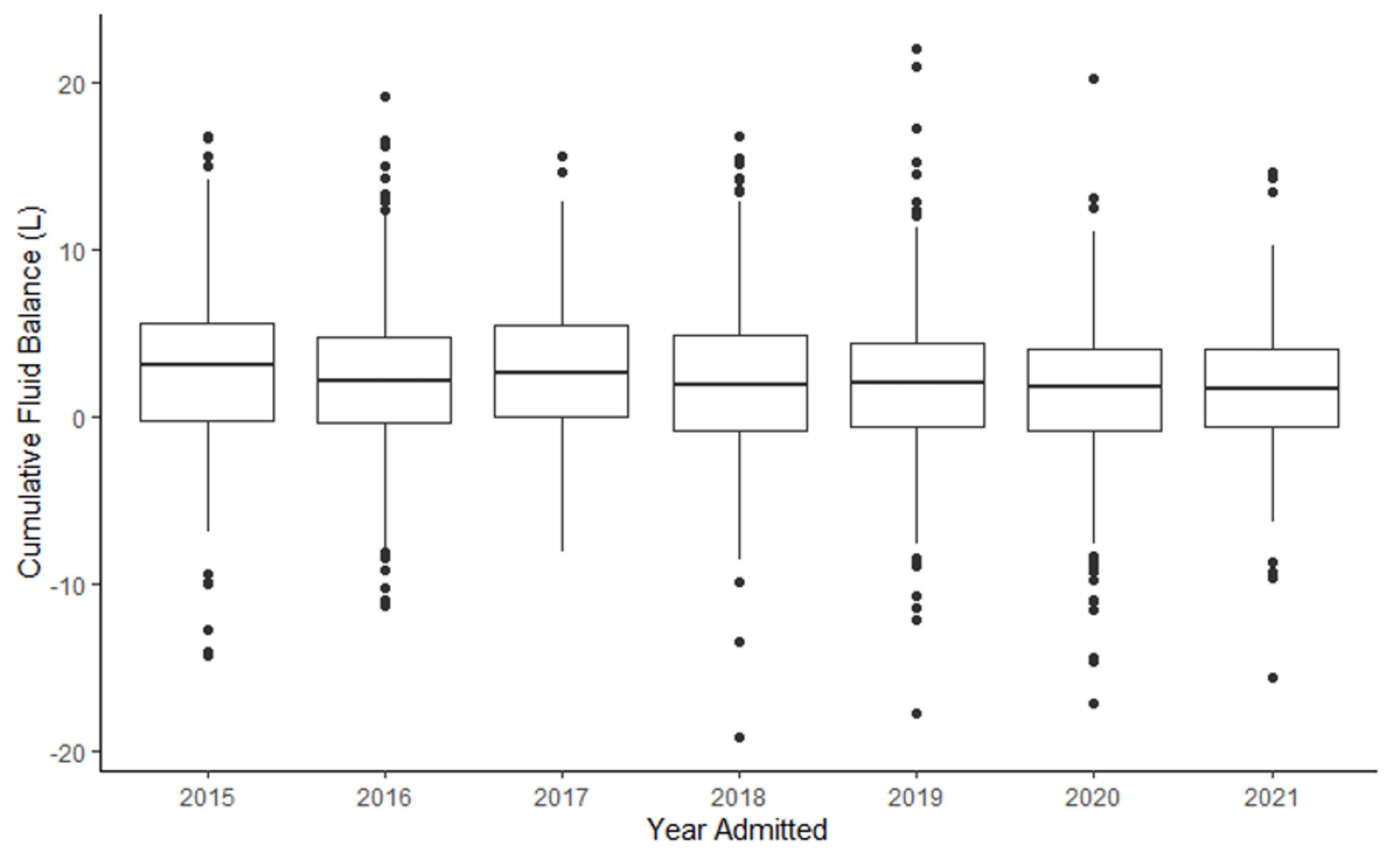
Box and whisker plots showing CFB trend over the duration of the study year by year Of Note: Year 2021 only includes admissions to June. CFB: cumulative fluid balance is in Liters (L)

The ANZROD risk of death, which is calculated from characteristics at admission and from physiologic disturbances within the first 24 hours of admission, did not vary significantly across the quintiles. However, APACHE III scores did vary among the quintiles and were highest in Q5 (p = 0.016). A temporal trend was observed over the period of the study, with more patients having a less positive fluid balance (Figure 3).

**Figure 4:**
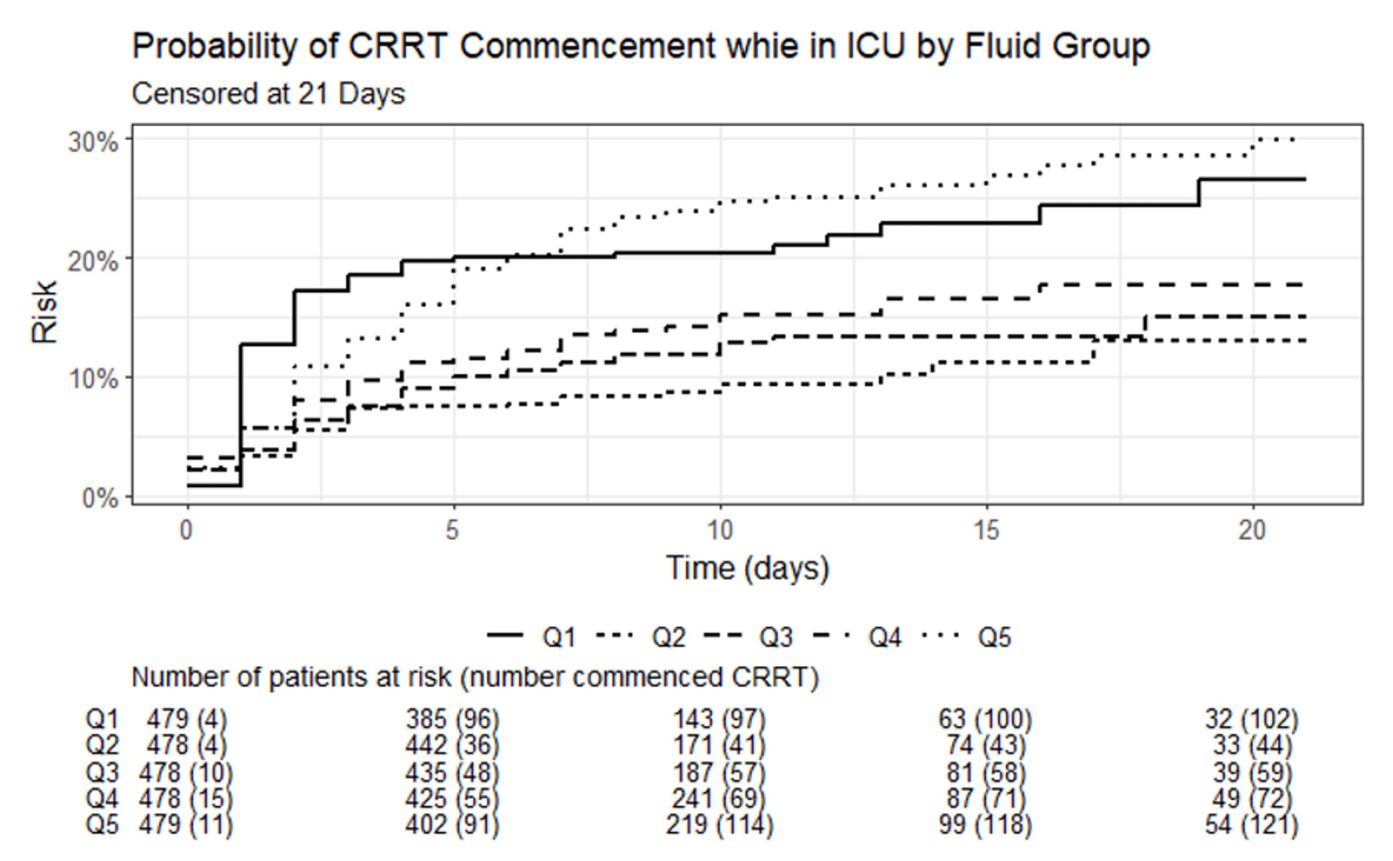
A Kaplan-Meier curve demonstrating the probability of patients requiring CRRT for each of the quintiles over 21 days in the ICU Day 0 represents patients with pre-existing dialysis requirements for end-stage renal disease. Each day starting from day 1 represents a 24-hour period per patient (e.g., if admitted at 15:00, days are from 15:00 to 14:59 the following day). CRRT: continuous renal replacement therapy; Q: quintiles, Q1 least fluid positive, Q5 most fluid positive.

Tables 5-6 show the duration of IMV and NIV across the CFB quintiles. Table 7 shows the mean duration of continuous renal replacement therapy (CRRT) across CFB quintiles. Figure 4 is A Kaplan-Meier curve demonstrating the probability of patients requiring CRRT for each of the quintiles over 21 days in the ICU, with the Q1 and Q5 having the highest probabilities. 

**Table 5:** Duration of invasive mechanical ventilation across the CFB quintiles CFB: cumulative fluid balance; L: liters; IQR: interquartile range; LWR: lower; UPRE: upper; SD: standard deviation; Q: quintiles; IMV: invasive mandatory ventilation; NIV: noninvasive ventilation

CFB (L)	Mean Days	SD	Median	Lower IQR	Upper IQR
Q1	12.17	15.14	9.33	5.06	15.02
Q2	8.88	8.28	7.36	4.71	10.71
Q3	7.15	5.39	6.17	4.54	8.75
Q4	7.36	5.58	6.25	4.58	9.04
Q5	8.54	6.96	7.08	4.83	10.56

**Table 6:** Duration of noninvasive ventilation across the CFB quintiles CFB: cumulative fluid balance; L: liters; IQR: interquartile range; LWR: lower; UPRE: upper; SD: standard deviation; Q: quintiles; IMV: invasive mandatory ventilation; NIV: noninvasive ventilation

CFB (L)	Mean Days	SD	Median	Lower IQR	Upper IQR
Q1	0.39	1.11	0	0	0.13
Q2	0.28	0.90	0	0	0.04
Q3	0.17	0.58	0	0	0.00
Q4	0.12	0.44	0	0	0.00
Q5	0.09	0.42	0	0	0.00

**Table 7:** The mean duration of continuous renal replacement therapy across the CFB quintiles CFB: cumulative fluid balance; L: liters; IQR: interquartile range; LWR: lower; UPRE: upper; SD: standard deviation; Q: quintiles; CRRT: continuous renal replacement therapy

CFB (L)	n	Mean CRRT Hours	SD	Median	Lower IQR	Upper IQR
Q1	103	63.63	30.60	65.0	43.5	86.0
Q2	46	58.61	31.44	64.5	34.0	88.0
Q3	44	54.25	31.37	55.0	27.5	73.5
Q4	45	47.98	28.42	50.0	28.0	71.0
Q5	82	51.07	31.15	54.5	22.0	75.0

**Figure 5:**
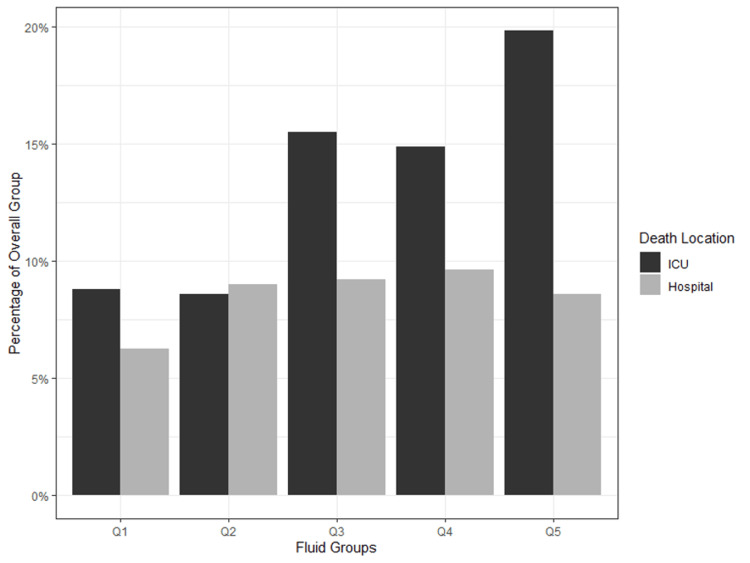
A bar graph showing the proportions of death for each quintile for both Hospital and ICU stay outcomes Q = Quintiles with Q1 being the most fluid negative group and Q5 being the most fluid positive group. ICU deaths are not included in hospital deaths.

**Figure 6:**
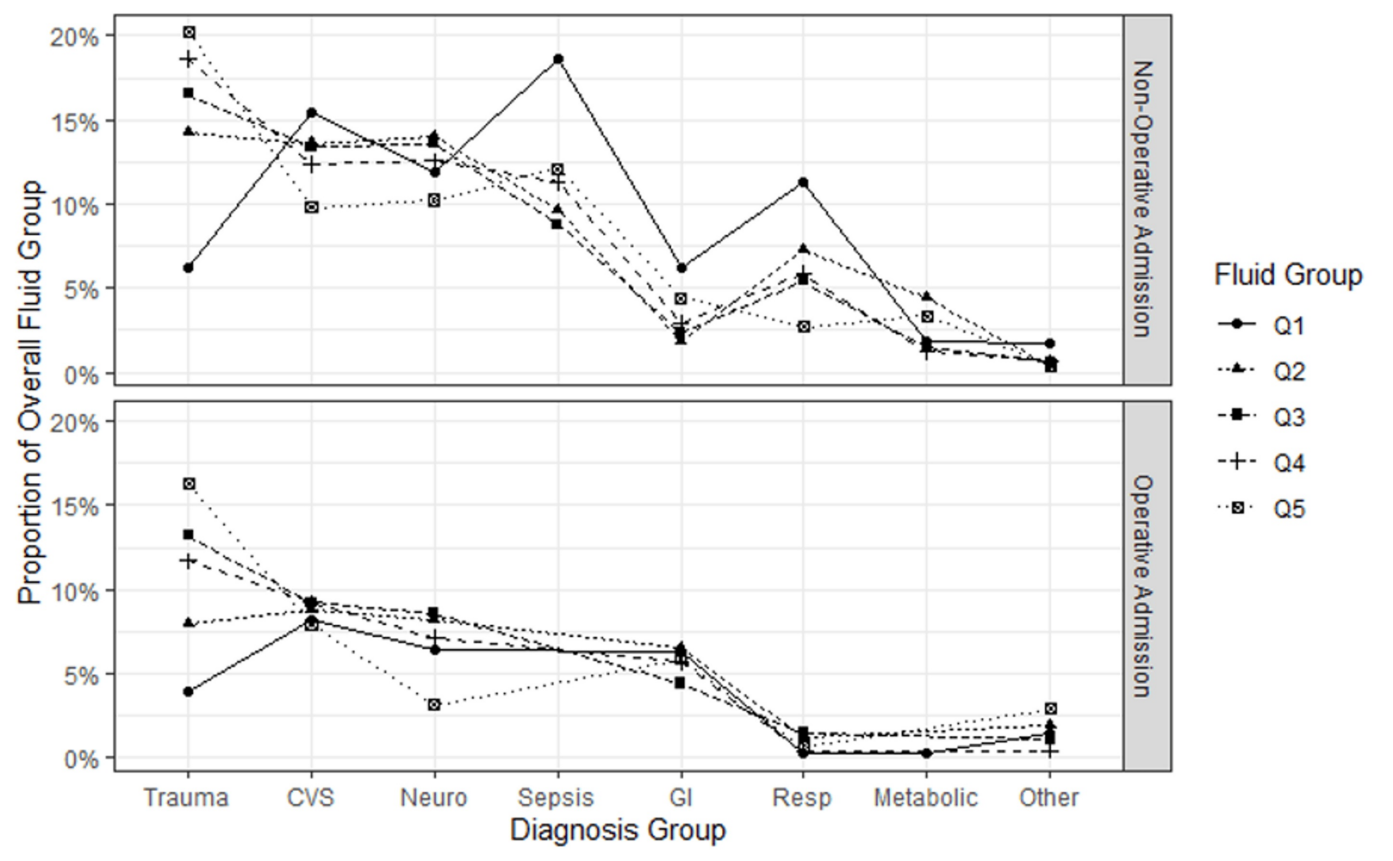
A line graph comparing the proportion of ANZICS diagnoses across the quintiles ANZICS: Australia and New Zealand Intensive Care Society; CVS: cardiovascular; GI: gastrointestinal; Neuro: neurological; Resp: respiratory; Q: quintile; Q1 least fluid positive, Q5 most fluid positive.

Figure 5 is a bar graph showing the proportions of death for each quintile for both Hospital and ICU stay outcomes. Figure 6 shows a line graph comparing the proportion of ANZICS diagnoses across the quintiles.

Primary outcomes

Hospital mortality increased progressively from 15.03% in Q1 to 28.39% in Q5 with statistically significant differences found among the quintiles (p < 0.00001). This is demonstrated in Table 8 alongside APACHE III and ANZROD predicted mortality rates for each of the quintiles. When actual mortality was standardized against APACHE III and ANZROD predicted mortality, it was lowest in the less fluid positive quintile (Q1), 30.54% (p< 0.01) and 23.51% (p<0.01), respectively. This then increased progressively in more fluid positive groups, with the highest mortality being in Q5 (the most fluid positive group), 32.19% (p = 0.078) for APACHE III and 24.39% (p = 0.043) for ANZROD.

**Table 8:** Hospital mortality analysis showing actual mortality compared to predicted mortality across the quintiles CFB: cumulative fluid balance, as median; L: Liters; ANZROD: Australia and New Zealand Risk of Death; AP3: APACHE III: Acute Physiology, Age, Chronic Health Evaluation; CI: 95% confidence interval; Q ( )– Quintile with median CFB in Liters *Estimates, confidence intervals, and p-values calculated per group using the two-sided exact binomial test. The risk of death for ANZROD and APACHE III was assessed with a pairwise Wilcoxon rank sum test with Bonferroni adjustment, which demonstrated no statistically significant difference between the fluid groups for predicted deaths but did find a statistically significant difference between Q1 and Q5 and Q2 and Q5 for actual mortality.

Fluid Group	n	Actual Mortality	APACHE III Predicted Mortality (%)	P-value	ANZROD Predicted Mortality (%)	P-value
		(95% CI)*				
Q1 (−3.26)	479	15.03 (11.95,18.55)	30.54	p <0.01*	23.51	<0.01*
Q2 (0.13)	478	17.57 (14.27,21.29)	27.69	p <0.01*	21.44	0.039*
Q3 (2.11)	478	24.69 (20.88,28.81)	30.56	p <0.01*	23.71	0.628*
Q4 (4.17)	478	24.48 (20.69,28.59)	29.58	p 0.014*	21.6	0.133*
Q5 (7.48)	479	28.39 (24.39,32.66)	32.19	p 0.078*	24.39	0.043*
Overall	2,392	22.03 (20.38,23.75)	30.11	p <0.01*	22.93	0.307*

**Figure 7:**
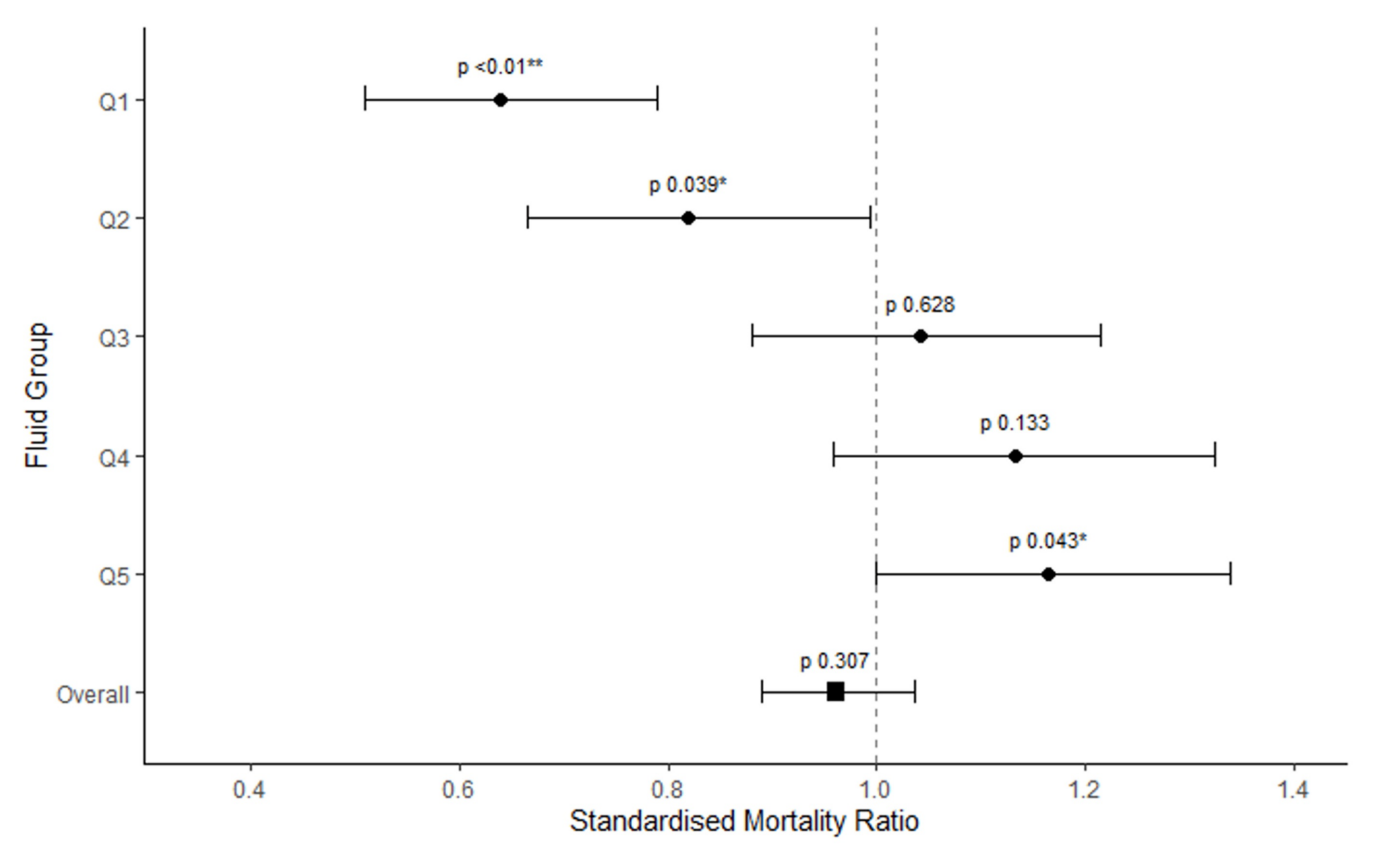
A forest plot of standardized mortality ratio (using ANZROD) for the cumulative fluid balance quintiles This Forest Plot demonstrates that Quintile 1 (the least fluid positive group) significantly favors survival while Quintile 4 and 5 (the more fluid positive groups) favor death. To the left of the vertical line on the x-axis, 1 favors survival. ANZROD: Australia and New Zealand Risk of Death; Standardized Mortality Ratio: actual/predicted mortality; Q: quintile; Q1 least fluid positive, Q5 most fluid positive

**Figure 8:**
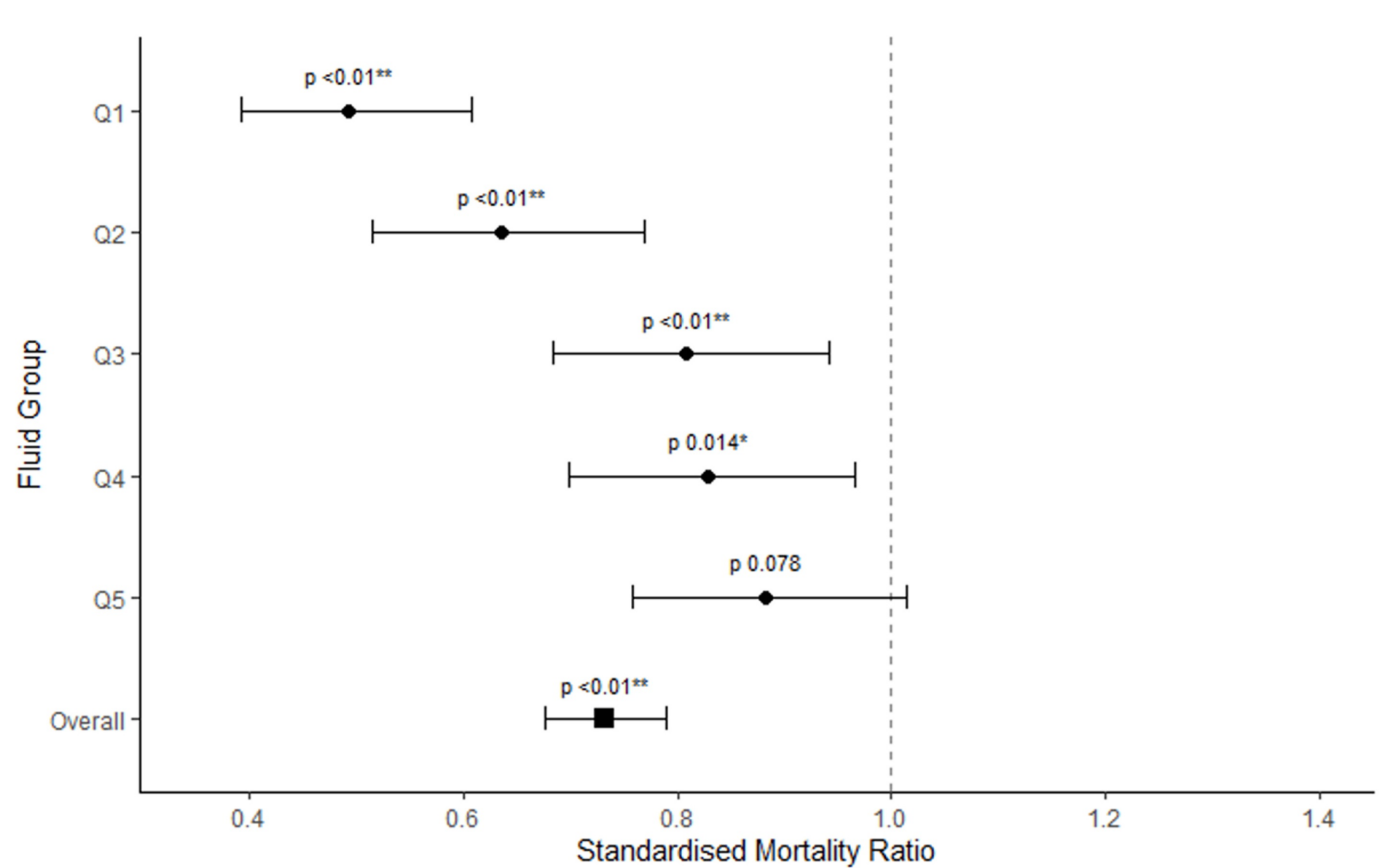
A forest plot of standardized mortality ratio (using APACHE III) for the cumulative fluid balance quintiles To the left of the vertical line on the x-axis, 1 favors survival. APACHE III: Acute Physiology, Age, Chronic Health Evaluation; Standardized Mortality Ratio: actual/predicted mortality; Q: quintile; Q1 least fluid positive, Q5 most fluid positive

Standardized Mortality Ratios along with their confidence intervals using both ANZROD and APACHE II are demonstrated in forest plots in Figures 7-8.

**Figure 9:**
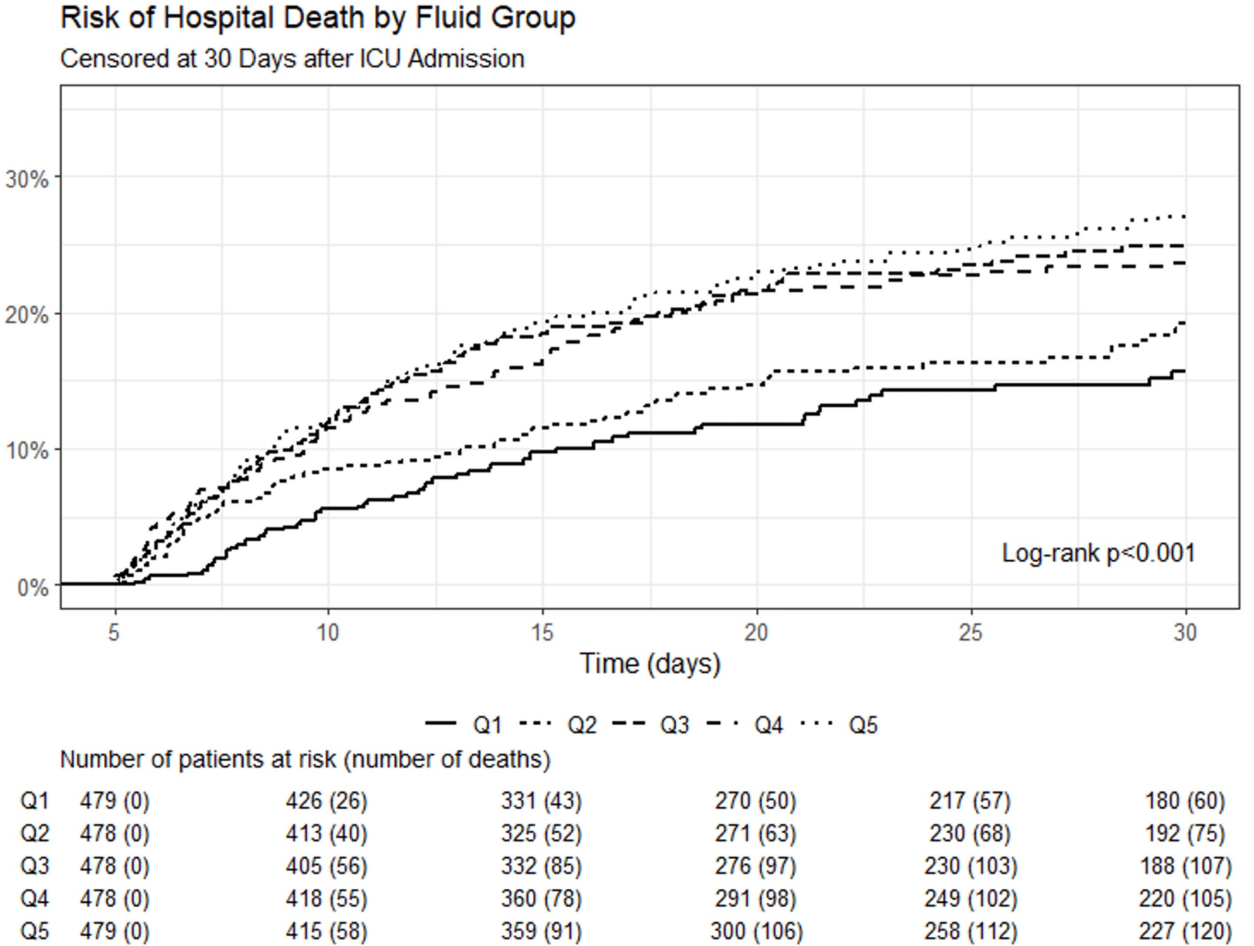
Kaplan-Meier Curve showing the risk of hospital death from day 5 out to 30 days Q: Quintiles with Q1 being the most fluid negative group and Q5 being the most fluid positive group.

Figure 9 demonstrates a Kaplan-Meier curve showing the risk of hospital death from day 5 (120 hours) out to 30 days of ICU admission, with a separation between the fluid groups, with the least fluid positive group having a lower mortality.

## Discussion

Key findings

In this retrospective cohort single center study, we analyzed 2,392 patient admissions and found that the less fluid positive patients had significantly lower mortality compared to those who were in the more fluid positive groups and this persisted despite adjusting for disease severity: actual mortality differed significantly from ANZROD and APACHE III predicted mortality, with the less fluid positive patients having lower actual mortality and the most fluid positive group having higher actual mortality than predicted.

The less fluid positive patients were both less likely to require vasopressors and received lower doses of vasopressors. They were also less likely to require IMV but more likely to receive NIV (although with greater mean duration). However, these patients had higher ICU and hospital stays. The high incidence of CRRT and more furosemide use in Q1 suggests the less fluid positive state was a result of deliberate clinical management targeting negative fluid balance in more patients within this cohort. Patients with sepsis were over-represented in this quintile, a group often requiring significant fluid resuscitation. This may be a result of this cohort being well resuscitated at presentation to the ICU. A temporal trend was also noted whereby patients had a less positive CFB in the later years.

We observed that the more fluid positive patients (such as in Q5) had higher APACHE III scores, were more likely to require and receive higher doses of vasopressors, had more severe kidney injury, more requirement for dialysis, and invasive ventilation. This is consistent with previous studies that have demonstrated the association between sicker patients, greater fluid resuscitation requirements, and adverse outcomes. Our findings differ from prior observational study data in that we corrected for disease severity (ANZROD).

Relationship to literature

To the best of our knowledge, there is a paucity in high-quality data in this area to guide clinical practice, particularly for those that have been corrected for standardized mortality rates, such as our study. Messmer et al in 2020 conducted a systematic review of observational studies with 31,076 patients in which they concluded that a more positive fluid balance (at day 3 of ICU admission) was associated with higher mortality [11]. Although several studies adjusted for disease severity in regression models, our study differed in that it adjusted for standardized mortality rates. Lee et al conducted a longitudinal analysis in 2015 on survivors of the ICU and concluded that positive fluid balance at the time of ICU discharge was associated with increased risk of death, after adjusting for markers of illness, in particular chronic health conditions such as underlying heart and kidney disease. In this study, compared to the lowest quartile of discharge fluid balance (median -1.5L), the highest quartile (median 7.6L) was associated with a 35% higher adjusted risk of death [12]. This differs from our study, which evaluated the effect of CFB on mortality 5 days (or 120 hours) into ICU admission and corrected for adjusted disease severity predictors.

Most of the literature involving mechanical ventilation and fluid balance revolved around acute lung injury (ALI), AKI, acute respiratory distress syndrome (ARDS), and coronavirus disease 2019 (COVID-19) in the more recent past: conditions that were not specifically evaluated in this study. Rosenberg et al, in a secondary analysis of a prospective cohort study (ARDSNET trial), showed that there was a mortality benefit in patients with ALI and ARDS who had a negative fluid balance by day four with the cohort divided into four groups (day 4 CFB < 0 L, 0 to 4.99L, 5 to 9.99L and > 10L) [13]. Van Mourik et al., in an observational cohort study, reported not only increased mortality but also duration of IMV with more positive fluid balance in ARDS [6]. In their analysis of data from the “Practice of VENTilation in COVID-19 patients (PROVENT COVID) study”, Ahuja et al in 2022 observed that those with higher CFB required a longer duration of IMV in COVID-19 patients with ARDS [14]. Our study observed that patients with respiratory diseases were over-represented in the less fluid positive quintile.

Wang et al performed a multicenter prospective epidemiological study involving 2,526 patients evaluating the impact of fluid balance on mortality in ICU patients with AKI and found that patients with AKI had high fluid balance and that fluid overload was an independent risk factor for the incidence and severity of AKI. They also found that a high CFB was associated with 28-day mortality following AKI [15].

Oh et al have demonstrated the negative effects of positive CFB on mortality in patients undergoing colorectal surgery [16]. Hatton et al and Elofson et al have investigated severely injured trauma patients and observed that those with positive fluid balance at 48 hours have an increased association with AKI and 7-day mortality [17,18].

The CLOVERS Trial investigators in their unblinded superiority trial investigating early restrictive or liberal fluid management for sepsis-induced hypotension concluded that the restrictive fluid strategy did not result in lower (or higher) mortality before discharge home at day 90 than the liberal fluid strategy in patients with septic shock [19]. The CLASSIC Trial group also concluded that fluid restriction in adults with septic shock did not result in fewer deaths at 90 days as compared to standard therapy [20].

POINCARE-2 was a stepped wedge cluster-randomized trial conducted to evaluate all-cause mortality in a broad range of critically ill patients by targeting fluid balance control two days into ICU admission. Both the intention-to-treat analysis and the as-treated analysis concluded that the conservative strategy was not associated with reduced mortality [21,22].

Interestingly, a post hoc secondary analysis of the STARRT-AKI trial reported regional practice variation for fluid balance management, with ICUs in Australia and New Zealand (ANZ) having a lower mean CFB (2211mL) at 14 days compared to ICUs in Europe (5641mL) and North America (7199mL). The ANZ ICU population had lower ICU and hospital mortality compared to other regions [23]. Wang and colleagues evaluated the impact of CFB on short- and long-term mortality in their retrospective study of critically ill patients and described increased 1-year mortality with positive CFB on days 4 to 7 of ICU admission [24].

Implication of study findings

Our results represent data on the effects of CFB from a tertiary critical care unit with a diverse case mix and showed that there is an association between survival and achieving a less positive CFB in ICU patients at 120 hours of ICU stay, which persists despite correction for severity of illness. However, any results of the study need to be interpreted with caution. While the study is supportive of the notion that a more positive fluid balance may be harmful, causality cannot be inferred. ICU patients are a heterogeneous population, and an individual patient’s optimal CFB is likely to depend upon many factors. Further evidence that helps clinicians determine when fluid administration may benefit an individual patient is required to guide practice.

Strength and limitations

There were several strengths to our study. It represents contemporary data from a study that was conducted on a large cohort of patients in a tertiary hospital ICU with a diverse case mix over six and a half years. Secondly, we adjusted our outcomes for the standardized mortality rate (ANZROD). Thirdly, the data was analyzed in a de-identified manner, which reduced the risk of bias.

There were some limitations. Firstly, this was a single-center study, conducted retrospectively. Although the case mix would be representative of the general Australian population and those in well-resourced countries, we acknowledge that the fluid management practices observed will mean the results are valid for the single institution and may not apply to units with different approaches. Secondly, some of the fluid balance data, such as gastric and drain outputs, were manually entered, and this could be a source of error. Thirdly, calculated CFB is not an exact measure, as the measurement of fluid output in patients with high insensible losses (e.g., losses to wound dressings) is not exact.

Fourthly, choosing a single time point for analyzing data at 120 hours into ICU admissions limits generalizability to shorter stay patients. This time frame was defined a priori. We aimed to include patients with a significant ICU stay and chose the time for CFB assessment of 120 hours to equal the inclusion criteria so as not to introduce a bias. Other studies have evaluated outcomes at day 3, 4, and at ICU discharge [8,9,10]. We acknowledge that this could introduce survivor bias by excluding earlier deaths and could limit external validity.

Fifth, our CFB does not include fluid resuscitation administered prior to or outside the ICU admission. Lastly, our adjustment for disease severity is unlikely to adequately account for the baseline differences that had evolved in our patients by 120 hours of their admission, with both APACHE III and ANZROD only considering data acquired up to the end of the first day of ICU.

## Conclusions

In this retrospective, single-center cohort study of critically ill patients, we observed that a more positive CFB at 120 hours following ICU admission was associated with increased hospital mortality. Notably, this association persisted even after adjustment for standardized mortality rates. These findings suggest that fluid accumulation may contribute to adverse outcomes, highlighting the potential clinical relevance of managing fluid balance. However, given the observational design and inherent limitations, further prospective studies are warranted to validate these results and to define optimal fluid management strategies in the critically ill population.

## Appendices

Calculation of Vasoactive Inotrope Score (VIS) - [6]

The peak mean vasoactive / inotrope dose ( in μg kg^−1^ min^−1^ or IU kg^−1^ min^−1^) over the first 5 days of ICU admission was used.

VIS = dopamine dose [μg kg^−1^ min^−1^]+dobutamine [μg kg^−1^ min^−1^] + 100 × epinephrine dose [μg kg^−1^ min^−1^] + 50 × levosimendan dose [μg kg^−1^ min^−1^] + 10 × milrinone dose [μg kg^−1^ min^−1^] + 10 000 × vasopressin [units kg^−1^ min^−1^] + 100 × norepinephrine dose [μg kg^−1^ min^−1^])
